# 2424: National trends in ambulatory Versus emergency department visits for low-income patients with skin and soft tissue infections

**DOI:** 10.1017/cts.2017.275

**Published:** 2018-05-10

**Authors:** Brianna M. D’Orazio, Joel Correa da Rosa, Jonathan N. Tobin

## Abstract

OBJECTIVES/SPECIFIC AIMS: Community-associated methicillin-resistant *Staphylococcus aureus* (CA-MRSA) skin and soft tissue infections (SSTIs) recurrence ranges from 16% to 43% and presents significant challenges to clinicians, patients, and families. The number of emergency department visits for SSTIs increased from 1993 to 2005 from 0.48 to 1.16 ED visits per 100 US residents (95% CI 0.94 to 1.39; *p*<0.001); high safety-net status EDs saw a 4-fold increase in visits. The CA-MRSA Project (CAMP2) comparative effectiveness research (CER) study aims to evaluate a home-based intervention implemented by Community Health Workers (CHWs) or “promotoras” to prevent recurrence and transmission of CA-MRSA in primarily low-income, minority patients presenting to primary care with SSTIs. The intervention disseminates and implements methods found effective in the REDUCE MRSA trial. The present analysis was conducted using publically available data set to characterize the national patterns of healthcare utilization for treatment of SSTIs. METHODS/STUDY POPULATION: An analysis was conducted using data downloaded from the CDC National Ambulatory Medical Care Survey (NAMCS) and the CDC National Hospital Ambulatory Medical Care Survey (NHAMCS) from 2012 (most recent data available) to evaluate the addition of Emergency Departments (EDs) as compared to Ambulatory Care as recruitment sources for a clinical trial to reduce CA-MRSA SSTI recurrence and household transmission. “Low-income” population was defined using “Expected Source of Payment” categories “Medicaid” and “Uninsured,” and ICD-9-CM dermatologic diagnosis codes for SSTIs and ICD-9-CM Procedure Codes for Incision and Drainage (I&D) were used to define a visit for SSTI treatment. RESULTS/ANTICIPATED RESULTS: In all patients, I&D was performed at a higher rate in EDs as compared with the ambulatory care setting (49.57 vs. 1.44 per 10,000 US residents in Medicaid and Uninsured; 44.48 vs. 5.24 per 10,000 US residents in all other insurance types). Nationally, low-income patients are 4 times more likely to have I&D procedure performed (OR 4.05, 95% CI 0.614–26.759, *p*<0.0001) and 5 times more likely to be diagnosed with an SSTI (OR 5.10, 95% CI 2.987–8.707, *p*<0.001) in the ED setting. DISCUSSION/SIGNIFICANCE OF IMPACT: These results confirm that low income patients seek primary care for SSTIs in both EDs and ambulatory care, such as Federally Qualified Health Centers (FQHCs). This also confirms the trend we have experienced in FQHCs in NYC, many of whom refer patients to the ED for the I&D procedure, and those patients return to the FQHC for follow-up. Thus, the most comprehensive test of using CHWs to disseminate and implement the findings from the REDUCE MRSA trial would engage both EDs and Ambulatory Care/FQHCs for patient identification and recruitment.

